# Processing Conditions, Rice Properties, Health and Environment

**DOI:** 10.3390/ijerph8061957

**Published:** 2011-06-03

**Authors:** Poritosh Roy, Takahiro Orikasa, Hiroshi Okadome, Nobutaka Nakamura, Takeo Shiina

**Affiliations:** 1 National Food Research Institute, National Agriculture and Food Research Organization, Kannondai 2-1-12, Tsukuba, Ibaraki 305-8642, Japan; E-Mails: okadome@affrc.go.jp (H.O.); nboy@affrc.go.jp (N.N.); 2 School of Food, Agriculture and Environmental Sciences, Miyagi University, 2-2-1, Hatatate, Taihaku, Sendai 982-0215, Japan; E-Mail: orikasa@myu.ac.jp

**Keywords:** rice processing, rice properties, CO_2_ emission, health, environment

## Abstract

Rice is the staple food for nearly two-thirds of the world’s population. Food components and environmental load of rice depends on the rice form that is resulted by different processing conditions. Brown rice (BR), germinated brown rice (GBR) and partially-milled rice (PMR) contains more health beneficial food components compared to the well milled rice (WMR). Although the arsenic concentration in cooked rice depends on the cooking methods, parboiled rice (PBR) seems to be relatively prone to arsenic contamination compared to that of untreated rice, if contaminated water is used for parboiling and cooking. A change in consumption patterns from PBR to untreated rice (non-parboiled), and WMR to PMR or BR may conserve about 43–54 million tons of rice and reduce the risk from arsenic contamination in the arsenic prone area. This study also reveals that a change in rice consumption patterns not only supply more food components but also reduces environmental loads. A switch in production and consumption patterns would improve food security where food grains are scarce, and provide more health beneficial food components, may prevent some diseases and ease the burden on the Earth. However, motivation and awareness of the environment and health, and even a nominal incentive may require for a method switching which may help in building a sustainable society.

## 1. Introduction

Rice (*Oryza sativa L.*) is the staple food for nearly two-thirds of the world’s population [1]. It has been reported that as much as 75% of the daily calorie intake of the people in some Asian countries is derived from rice [2]. It is reported that the world's stocks of stored rice grain have been falling relative to each year's use, because the consumption surpasses the production [3]. The deficiency of food production not only increases the food price, but also increases malnourished population in the world might result different social and economic unrest. Researchers have also predicted that the global warming will make rice crops less productive [4–7] and it could threaten to erase the hard-won productivity gains that have so far kept the rice harvest in step with population growth. Rice processing is a combination of several operations to convert paddy into well milled silky-white rice, which has superior cooking quality attributes [8]. The majority of consumers prefer well milled rice (WMR) with little or no bran remaining on the endosperm. It has also been reported that consumer preferences vary from region to region. For instance, the Japanese like well milled sticky rice [9], but Americans prefer semi-milled long grain or even brown rice (BR), whereas people in the Indian sub-continent prefer well milled parboiled rice [10].

Rice properties are known to be dependent on the variety of rice, methods of cultivation, processing and cooking conditions. Although the parboiling treatment helps in retaining some of the nutrients, reduce breakage loss during milling and increase head rice recovery (whole rice kernels after milling), consumed a considerable amount of energy and labor. It has also been reported that the nutritional value and head rice recovery reduce with the higher degree of milling (DOM). Usually, 10% by weight of brown rice is removed (outer layer) during milling, with the lower values occurring in countries where the DOM is regulated [11]. Rahaman *et al.* [12] reported that milling should be restricted to 7 to 8% for maximum recovery, which is the common practice in most developing countries including Bangladesh. In India, it is restricted to 4% by weight of BR [13]. The proteins, fats, vitamins, and minerals are concentrated in the germ and outer layer of the starchy endosperm [11,14] and these are removed by milling operation. The growing health consciousness led consumers to start consumption of rice milled to lower degrees (partially-milled rice: PMR) or even BR in some countries. In addition various value-added rice products (germinated brown rice: GBR), rice bread (RB) *etc*.) have also been developed. Rice properties and the environmental impacts of rice are known to be dependent on the variety of rice, methods of cultivation and processing conditions, and consumption patterns. Hence, the authors are intended to discuss rice processing, physicochemical properties of rice, and their impact on food security, human health and the environment.

## 2. Rice Processing

### 2.1. Parboiling

**Figure 1.**
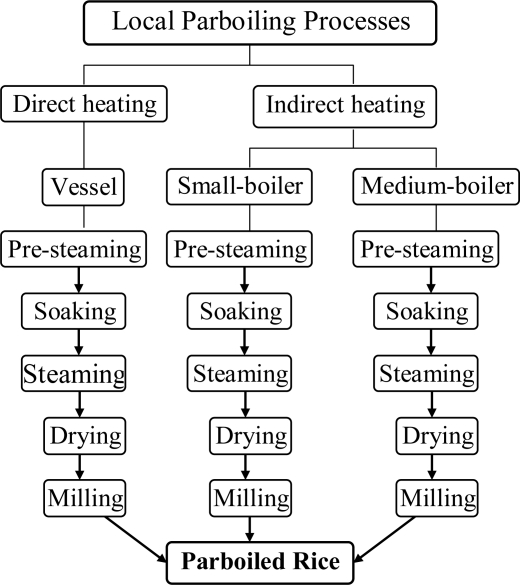
An overview of local parboiling processes [35].

Parboiling is an ancient method of rice processing, widely used in the developing countries like Bangladesh, India, Sri Lanka, and in some rice exporting countries. Parboiled rice (PBR) has been produced by both traditional and modern methods. Various parboiling devices and techniques have been developed [12,15–32]. Modern methods are energy and capital intensive, and are not suitable for small-scale operation at village level [25,33]. The local parboiling device consists of pottery to boiler, used for direct or indirect heating and single or double steaming, which consume a different amount of energy [34,35]. Agri-residues are the main sources of energy for local parboiling, especially the residues of rice processing industries. However, sun drying is the common practice in local parboiling processes. A nearby pond, river, lake or tube-well is the source of the water for parboiling. Figure 1 shows an overview of local parboiling processes.

Parboiling treatment induces various physicochemical changes in paddy which play an important role in storage, milling, cooking and eating qualities [36–46]. Although PBR is known to have a number of advantages, require more energy, water and time for processing and cooking [35,47–51] than the untreated rice. The parboiling treatment gelatinizes the rice starch, improves the hardness of the rice upon drying, minimized the breakage loss and thus increases the milling yield [42,45,52–54]. Over-parboiling resulted in over-opening of the husk components followed by bulging out of the endosperm which initiates surface scouring during milling and the resultant ground particles being lost into the husk and bran. However, incomplete or non-uniform parboiling produces white-bellied rice, which breaks easily during milling, and reduces the head rice yield [22,55]. The parboiled rice produced in the boiler processes is considered to be suitable and better because it has greater customer acceptance and market value, but require a greater initial investment [35]. The quality of PBR depends on the paddy, intensity of parboiling, drying condition and moisture content after drying, DOM and the milling devices [34,40,44,54,56–58].

### 2.2. Dehusking and Milling

Dehusking and milling process removes the outer part of paddy (husk and bran) to make it edible. There are three main types of husking machine, which includes: stone dehullers, rubber roll and impeller type husker [59]. Stone de-hullers are still common in tropical Asia, where BR is immediately milled with either an abrasive or friction mill. It has been reported that types of liner significantly affect the husking performance [60]. It has also been reported that the Engleberg-type or steel hullers are no longer acceptable in the commercial rice milling sector, as they lead to low milling recovery and high grain breakage [61]. Abrasive or friction type milling machines are used to remove the bran. It has been reported that the abrasive mill can over-mill readily. In the Engelberg or huller type mill, dehusking and milling are performed in one step with greater grain breakage. Using a dehusker before milling improves both the milling and head rice yields. During parboiling treatment the husk splits and loosen, which makes it easier for dehusking [24,47]. The energy consumption during dehusking process is also induced by the severity of steaming treatment and it tended to have decreased with the increase of steaming time [34].

The DOM is a quantification of the amount of bran that has been removed from kernels during the milling process. The DOM is influenced by the grain hardness, size and shape, depth of surface ridges, bran thickness and milling efficiency [62]. Harder rice requires greater energy to obtain the same DOM [34,62]. The energy consumption during milling is also depends on the grain thickness, hardness, shape and variety, and DOM [34,62–65]. Lower surface hardness facilitates breakage during milling, resulting lower milled rice recovery and quality [66] in the case of long grains compared to that of short grains. Mass loss and breakage are affected by cultivar, kernel shape, and thickness of aleurone layer [67,68]. The flow ability of short grains is higher than that of long grains through the milling chamber of friction type milling machine results in lower degree of breakage during milling [12], leads to produce greater amount of head rice. Roy *et al.* [51] reported that in the case of the milled rice option, PBR contains 3% broken grains and untreated rice contains 13% broken grains, would be acceptable to the local consumers. The overall energy consumption during dehusking and milling is reported to be greater in the case of PBR compared to the untreated rice [34].

**Figure 2.**
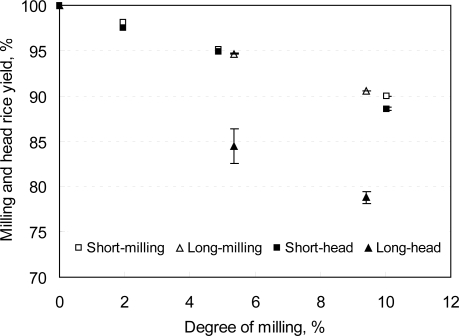
Effect of degree of milling on milling and head rice yield [65].

The properties of rice desired by consumers include whiteness, translucency, low percentage of damaged or broken grains and low foreign matter. The amount of bran in rice kernels varies with variety, environmental conditions and agronomic practices [66,69]. The bran is reported to be darker than the polish [11]. In the case of untreated rice, head rice yield decrease and lightness increases with increase in DOM [58,65,70]. The BR of Japonica variety has greater color intensity than that of Indica variety [65,69]. For a certain DOM the difference in milling yield between short and long grains is reported to be insignificant (Figure 2).

### 2.3. Germinated Brown Rice

Brown rice kernels soak in warm water (30–40 °C) until they just begin to bud (exact time depends on water temperature), which is known as germinated brown rice (GBR). The potential health benefits and superior quality of GBR have attracted public attention and it becomes a popular healthy food, and different local governments (prefecture) in Japan are promoting the GBR. As a result of its popularity, modern rice cooker has also been developed to facilitate the production of GBR at households with various time spans (6–15 h). In this process, washed BR used to put on the rice cooker vessel with adequate amounts of water. Roy *et al.* [71] had used 2.9 L water for 750 g of rice in the germination process. The water temperature in the rice cooker during germination process is reported to be about 31 °C [72]. The germinated rice washes several time to avoid off odor, hence more water is required compared to other form of rice [72].

The GBR contains much more fiber, essential amino acid, lysine, and gamma-aminobutyric acid (GABA) than conventional BR. GBR contains more nutrients compared to the milled rice *i.e.*, 10 times GABA, and about 4 times dietary fiber, vitamin E, niacine and lysine, and about 3 times B_1_, B_2_ and Magnesium [73]. Saikusa *et al.* [74] reported that γ-amirobutyric acid (GABA) increased dramatically if BR is soaked in water at 40 °C for 8–24 h. The phenolic compounds are also reported to be more abundant in BR and GBR [75]. These nutrients accelerate the metabolism of brain help in preventing major diseases such as gastrointestinal cancers, heart disease, high blood pressure, diabetic and beriberi, constipation, and Alzheimer’s diseases [73,76–79]. Jeon *et al*. [80] concluded that GBR may be effective for suppressing liver damage. GBR also enhances maternal mental health and immunity during lactation [81]. Okada *et al*. [82] reported that intake of GABA for 8 consecutive weeks suppressed blood pressure and improved sleeplessness, and autonomic disorder observed during the menopausal or presenile period.

### 2.4. Hydration and Cooking

Milled and BR exhibit different rates of moisture absorption [65,83] with milled rice initially absorbing water at a faster rate than BR because the milling treatment removes the outer protective layers of rice caryopsis, the endosperm of milled rice becomes relatively prone to water absorption [84]. On the other hand, wax content in the BR seed coat and on the pericarp reduces water absorption [85]. Roy *et al.* [65] noted that the difference in the rate of water absorption is insignificant among the rice milled to different DOM, which supports the earlier findings that removing 1% of the outer BR kernel increases the water absorption to that of highly milled rice [86]. The final moisture distribution in BR is reported to be more homogenious than in milled rice [87]. It is also reported that approximately 24% moisture evenly distributed throughout the grain is enough to bring out its gelatinization after normal atmospheric steaming [19,26,88] which indicates that 1 h of soaking might be enough to facilitate the cooking process of rice [65]. The gelatinized starch hydrates more compared to raw starch [89]. The PBR absorbs water at a faster rate and to a higher extent at low (room) temperature and it is proportional to the severity of heat treatment [16,49,90,91]. If water absorption during this process is insufficient, the starch in the central part of the grain does not fully gelatinize and swell, resulting in cooked grains with a hard texture [92].

The presoaking treatment improves the moisture content (MC) of rice, appears to cause quick heat transfer during cooking and thereby reduces the cooking time [49,91,93]. The functionality and sensory properties of rice are also reported to be affected by the DOM, processing conditions and the variety of rice. It is also reported that larger and thicker grains require longer cooking times [94,95]. Milling reduces the kernel size and gelatinization temperature (GT) to about 20% DOM [96], possibly leading to decrease in preset cooking times for various forms of rice. The thermal properties of rice are reported to be dependent on the variety and processing conditions which affects the cooking properties of rice [96–101]. Physicochemical properties, such as MC, adhesion and hardness, cooking time, energy consumption are induced by the processing conditions which affect the eating quality of rice. The stickiness of PBR decreases due to parboiling treatment and depends on the severity of treatment [49,102–104]. The hardness of cooked rice is reported to be dependent on the MC of cooked parboiled and untreated rice [49,65,104,105]. Roy *et al.* [106] noted that the hardness and adhesion of cooked rice were dependent not only on the MC but also on the forms and variety of rice. The MC in cooked rice for acceptable soft texture (66–69%: [49]) or optimally cooked rice (about 75%: [94]) varies widely. Also, the PBR requires a greater water-rice ratio during cooking than that of untreated rice. Rice with low amylose content is generally soft when cooked, whereas rice with high amylose content has higher grain hardness [107]. High-amylose rice has more very long chains than low-amylose rice [108,109]. The more long chains, the firmer is the rice when cooked and vice-versa [110]. Rice with high water binding capacity normally yields soft texture cooked rice [95]. At the same level of MC, cooked PBR is reported to be harder than the cooked untreated rice [91,102]. Cooked PBR retains better shape, reduced stickiness, and lesser solids losses into the cooking gruel, if cooked in excess water [24,111]. The loss depends on the severity of parboiling [94,104,112].

Rice contamination is reported, if contaminated water is used for cultivation, processing and cooking [113–126]. Highly significant genetic differences in arsenic accumulation during rice cultivation were detected in the arsenic contaminated area in Bangladesh. The contamination was higher for the traditional variety (Bangladeshi landraces) compared to that of improved BRRI cultivars. The rate of contamination was also dependent on the concentration of arsenic in water used for irrigation [126]. The *boro* (grown in summer: November–June) had greater total arsenic compared with *aus* (prekharif: April–September) and *aman* rice (irrigated by rain water, kharif: June–December) which was related to the use of higher volume of arsenic polluted ground water for irrigation [121]. The arsenic contamination also depends on the processing conditions of rice. Higher contamination was noted for the parboiled rice compared to that of untreated rice, where contaminated water was used for parboiling treatment. Final arsenic concentrations were reported to be 332 and 290 μg/kg in raw parboiled and untreated rice, respectively [122].

Arsenic retention in cooked rice is dependant on the type and variety of rice, cooking method, arsenic concentration in cooking water, if arsenic contaminated water is used for cooking [115,120–124,127,128]. Arsenic retention in rice varied from 45–107% of the arsenic in cooking water [113,116,118,124]. On the other hand, Devasa *et al.* [120] noted that all the arsenic present in cooking water may be retained during boiling of rice, increasing the contents of this metalloid to significant levels from a toxicological viewpoint. Bae *et al.* [113] reported that the arsenic retention in cooked rice is also dependent on the water absorption of rice. Roychowdhury *et al.* [117] confirmed that total arsenic content increases where the water used for cooking had high arsenic contents. The arsenic retention in cooked rice is reported to be lower in excess water cooking method compared to that of optimum water [121,128]. Sengupta *et al.* [116] described that when the rice (four different varieties) is cooked in an excess of water and the remaining water is then discarded the retention of arsenic is less (43%) than that observed in optimum water cooking (no cooking gruel) preparations (72–99%).

In excess water cooking method arsenic concentration in cooked rice (BRRI 28, parboiled) and in gruel were 0.40 ± 0.03 and 1.35 ± 0.04 mg/kg, respectively. In the case of non-parboiled (untreated) rice, arsenic concentration in cooked rice and in gruel was reported to be 0.39 ± 0.04 and 1.62 ± 0.07 mg/kg, respectively. Arsenic concentrations in parboiled and untreated rice were 0.89 ± 0.07 and 0.75 ± 0.04 mg/kg, respectively, if cooked in optimum water (no cooking gruel). However, no significant difference was reported between cooked parboiled and untreated BRRI hybrid rice in optimum water cooking methods [115]. It seems that non-contaminated water was used for parboiling treatments in that study, hence no difference in arsenic content between raw parboiled and untreated rice was reported. Rinse washing and high volume of cooking water is reported to be effective, if distilled water is used. High volume excess water cooking method removed 35–45% arsenic that of uncooked rice [128]. The rinse washing was effective for removing about 10% of the total and inorganic arsenic from the basmati rice, but less effective for other types of rice. Hence, it seems that PBR becomes relatively prone to arsenic contamination compared to that of untreated rice, if contaminated water is used for parboiling and cooking. Arsenic contamination could be avoided or controlled, if contaminated water is not used in rice cultivation, processing and cooking.

## 3. Food Components, Health and Environment

Kernels that have been milled to a different degree reportedly have varying functional and sensory properties [129,130]. Protein concentration is highest on the surface and gradually decreases toward the center of the kernel [58,131], however, starch concentration increases from the surface to the core of the milled rice. The germ and bran contain high level of minerals, protein and vitamins. The removal of the germ and bran from the BR produces milled rice which contains less food nutrients compared to that of BR (uncooked). Water soluble nutrients disperse into the endosperm but fat moves out during parboiling treatment, hence parboiled milled rice contains more water soluble nutrients and lesser fat for a certain DOM [132,133]. All the thiamine is removed from the untreated BR but only half from parboiled, if 10% outer portion is removed [134]. On the contrary, total thiamine and nicotinic acid content in BR are reported to be reduced after parboiling [134,135]. The protein content of brown or milled rice noted to be unaffected by parboiling [41]. The solubility of protein decreases after parboiling [41,136]. The riboflavin content also remains unaffected [137,138]. The energy and protein contents were estimated to be increased, and the lipid and dietary fibers decreased with the increase of DOM. The higher MC yields greater amount of cooked rice, might offset the difference in the energy and the protein contents [65,71,106]. Table 1 shows food components in different forms of rice. Energy content in Japonica rice is greater compared to the Indica rice might be because of lower MC, which indicates that for a certain amount of energy intake, greater amount of cooked Indica rice need to be consumed compared to that of Japonica.

Agriculture provides the nutrients essential for human life. Insufficient output of even one essential nutrient over a long time will create several social and human health problems [139]. McCarrison and Norris [140] concluded that beriberi is associated with the consumption of untreated rice, but not with the PBR. It is also noted that incidence of beriberi drops dramatically when untreated rice diet changed to PBR [141]. The presence of the amylose-lipid complex II may contributes to the low postprandial glucose increments, since these complexes has melting temperature above 100 °C and, therefore, are not melted during the cooking of neither the untreated nor the PBR samples. Most rice used for parboiling has intermediate (20–25%) or high (>25%) amylose content [142]. It has noted that high amylose content lowers the starch digestion rate, measured as the glycaemic or insulinaemic response to rice [143–146]. It is noted that parboiling treatment lowers the glycaemic response to rice and it depends on the severity (intensity) of parboiling [147,148]. In contrast, no difference was also reported [145,146]. It seems that not only parboiling but also the variety and forms of rice play an important role to glycaemic response of rice. The growing health concern leads humans to diversify their diet and helps to avoid some of the diet related diseases. The enrichment and fortification of cereals and other foods made a significant impact on the prevention of disease [149] and the development of functional foods may also play a key role in controlling/avoiding the diet related diseases.

**Figure 3.**
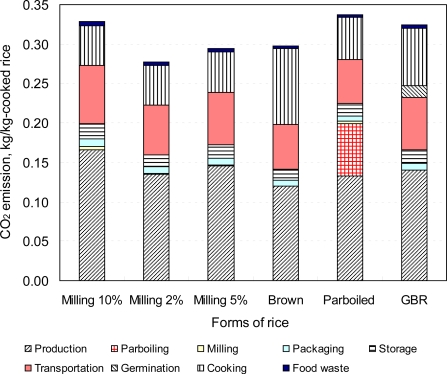
Life cycle CO_2_ emission of different forms of rice.

The environmental impacts of rice depends on the location of cultivation, variety, processing and forms of rice [50,51,71,150,151]. It is reported that half of the world’s households cook daily with biomass energy. Domestic cook-stoves—either traditional or improved—used for cooking in rural areas cause incomplete combustion of biomass and emit gaseous substances and suspended particulates which are responsible for many health problems [152,153]. Women who do the cooking are exposed to the domestic smoke and also the children, particularly infants and young children, who spend most of their time around their mother. PBR requires longer cooking time than that of untreated rice [49,94]. It seems that longer cooking time not only consumes greater energy but also forces them (who do the cooking) longer exposure to the domestic smoke than that of untreated rice may have greater health risk in the rural area of the developing countries. The untreated process is noted to be both the environmentally sustainable and cost effective process compared to the PBR, if milled rice is consumed instead of head rice [51]. Figure 3 represents the life cycle emission of different forms of rice [*Koshihikari & Basmati* (parboiled)]. The life cycle emission was found to be the highest for the PBR, followed by the GBR, and the lowest was the PMR (milling, 2%). Emissions were greater for BR than those of WMR and PMR, even though no milling is required because more energy is consumed during cooking compared to the others. The environmental load of PMR rice was found to be slightly lower than that of WMR because of the difference in head rice yield, which leads to lower production stage emissions. It is also noted that a change in rice consumption patterns would reduce 2–16% of CO_2_ emissions from the life cycle of rice [71,72].

The PBR also requires more water than that of PMR, BR and untreated rice because a considerable amount of water is used during soaking and steaming. The GBR also requires more water than that of PBR because of the huge amount of water requirement during germination and washing the GBR (to control odor) before it is cooked or packed [106]. Although the effluents discharged from local rice mills do not contain any toxic compound or pathogenic bacteria, repeatedly discharging them into the open may become a public health hazard, as the stagnant water not only encourages a variety of organisms but also emits off-odors in and around the local area. Relatively higher population of total aerobic bacteria, staphylococci, lactic acid bacteria and yeast were reported in socked water of the cold soaking method than the others. The modern rice mills adopting hot soaking and where there is a continuous discharge of effluents, with a high chemical oxygen demand (COD), phenols and sugars, into a localized area may have environmental concerns [154]. Table 2 shows the biochemical characteristics of soaked water from different parboiling methods. Growth of the parboiling sector or GBR sector may lead to water shortages and contamination. Washing is a common practice in the cooking process of rice. Biological oxygen demand (BOD) and COD in washed water known to be higher for milled rice compared to that of BR or PBR. Total BOD and COD in washed water produced by washing of 166 g regular rice (WMR) are reported to be 2,685 and 1,207 mg, respectively (http://www.musenmai.com/nichido.html).

It seems that the PMR may not only reduce the environmental load but also improves the head rice yield than that of WMR. Although the GBR consume greater amount of water, the BR, PMR (DOM 2%) and GBR option would improve food security in the regions where food is scarce, especially in developing countries. Moreover, PMR, BR and GBR provide various health benefits beyond their nutritional values. Considering their environmental load, it would be wise to consume the PMR than others. However, the taste of rice and its acceptability need to be considered to adopt lower DOM.

## 4. Discussion

The combination of population growth and economic development with the decreasing per capita land area, puts a great stress on arable land, water, energy and biological resources. The necessary expansion of global food production is becoming increasingly difficult to achieve. Water scarcity together with soil erosion, land degradation and climate change are the main threats to future increase in productivity. It is also noted that the climate change will make rice crops less productive [4–7] and it could threaten to erase the hard won productivity gains that have so far kept the rice harvest in step with population growth. As rice consumption surpasses production, the world's stocks of stored rice grain have been falling relative to each year’s use [3]. The deficiency of food production not only increases the food price, but also will increase malnourished population in the world might cause social and economic unrest. It is reported that 864 million people were under nourished worldwide in 2002–2004 [155].

The standard of rice and consumption preferences varies from country to country and region to region. Although the economic value of rice depends on the percent of whole milled grain production, the production cost, rice yield and food components in rice depend on the forms of rice [51,71]. The world paddy production is reported to be 685 million tons in 2008 [156]. It would produce about 541, 530, 514, 498 and 487 million tons of rice, if DOM is restricted to 0, 2, 5, 8 and 10%, respectively [72]. A change in consumption pattern from PBR to untreated rice, WMR to BR which contains more health beneficial food components may conserve about 43–54 million tons of rice and reduces the risk of rice contamination and prevent some diseases. It would be very helpful in the countries or region, where food grains are scarce and abate the malnourishment. Most of the milling facilities in the local area produce a byproduct as a mixture of the husk, bran and broken grains and used as poultry or animal feed, or as a solid biomass fuel in the rural area of the developing countries, have to be considered for any such change. However, considering the scarcity of food grains, and energy consumption and environmental pollution, it would be wise to consume untreated PMR, if consumers are satisfied with taste. Adoption of PMR not only ease the burden of the Earth but also provide more health beneficial food components for humans well being. Moreover, motivation and awareness of the environment and health, and even a nominal incentive may require for such a method switching.

## 5. Conclusions

This study reveals that the physicochemical and thermal properties, and environmental load of rice depend on the production and processing conditions. The BR, GBR and PMR contain more health beneficial food components than the WMR, which help to avoid different diseases. A change in consumption patterns from PBR to untreated rice, and WMR to PMR may conserve a considerable amount of grains. A switch in production and consumption patterns would improve food security where food grains are scarce, and also provides more health beneficial food components and ease the burden of the Earth. Although the arsenic concentration in cooked rice depends on the cooking methods, parboiled rice (PBR) seems to be relatively prone to arsenic contamination compared to that of untreated rice, if contaminated water is used for parboiling and cooking. However, consumer preference and taste of rice have to be considered for a method switching.

## Figures and Tables

**Table 1.** Food components in different forms of cooked rice (hardness of cooked rice, 10 N) [72].

**Variety/forms of Rice**	**DOM, %**	**MC, % (w.b.)**	**Food components and energy content per 100 grams cooked rice**				
			**Carbohydrate, g**	**Dietary fiber, g**	**Protein, g**	**Lipid, g**	**Energy, kJ**
Koshihikari	0.0	69.1	26.1	1.3	2.1	1.0	521.0
	2.0	66.3	29.3	1.1	2.2	0.8	565.8
	5.0	64.8	31.5	0.7	2.3	0.6	590.6
	10.0	61.5	35.6	0.5	2.5	0.4	649.6

IR28	0.0	72.5	22.0	1.3	2.8	0.9	458.5
	5.4	72.0	24.0	0.7	2.7	0.3	465.4
	9.4	71.5	24.7	0.7	2.8	0.2	475.5

Germinated brown rice	-	63.7	30.6	1.7	2.5	0.9	604.5
Parboiled (Basmati)	-	70.1	26.4	0.5	3.1	0.3	505.5

**Table 2.** Biochemical characteristics of soaked water of different parboiling methods [154].

**Source of soaked water**	**Soaking time, h**	**Components, mg/L**				
		**Total sugar**	**Amino nitrogen**	**Total phenol**	**BOD**	**COD**
Household	0	4	0	3	140	209
	12	10	17	4	206	306
Cold-soaking	0	4	0	3	140	209
	72	47	61	10	1039	1238
Double-steaming	0	22	9	19	117	244
	24	63	78	48	293	765
Hot-soaking	0	34	0	4	129	260
	4	641	104	62	30	2491
Pressure-parboiling wash water	-	1	7	20	57	198
